# Correction: Engineering the morphology of palladium nanostructures to tune their electrocatalytic activity in formic acid oxidation reactions

**DOI:** 10.1039/d2na90049a

**Published:** 2022-06-21

**Authors:** Bulti Pramanick, Trivender Kumar, Aditi Halder, Prem Felix Siril

**Affiliations:** School of Basic Science, Indian Institute of Technology Mandi Himachal Pradesh 175005 India prem@iitmandi.ac.in aditi@iitmandi.ac.in

## Abstract

Correction for ‘Engineering the morphology of palladium nanostructures to tune their electrocatalytic activity in formic acid oxidation reactions’ by Bulti Pramanick *et al.*, *Nanoscale Adv.*, 2020, **2**, 5810–5820, https://doi.org/10.1039/D0NA00798F.

The authors regret mistakes in Fig. 9, where the XPS curve corresponding to Pd_0D_ was reported as Pd_1D_ and the XPS curve corresponding to Pd_1D_ was reported as both Pd_0D_ and Pd_2D_.

**Fig. 9:**
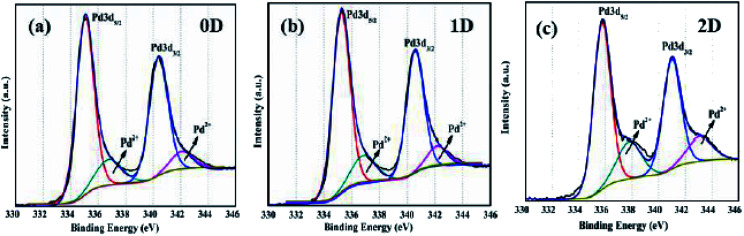
High resolution X-ray photoemission spectra of Pd nanostructures corresponding to Pd 3d of (a) 0D, (b) 1D and (c) 2D.

The XPS curves have been replotted from the original raw data and are shown here.

An independent expert has viewed the corrected images and associated raw data and has concluded that they are consistent with the discussions and conclusions presented.

The Royal Society of Chemistry apologises for these errors and any consequent inconvenience to authors and readers.

## Supplementary Material

